# Metabolic Interdependency of Th2 Cell-Mediated Type 2 Immunity and the Tumor Microenvironment

**DOI:** 10.3389/fimmu.2021.632581

**Published:** 2021-05-31

**Authors:** Simon Schreiber, Christoph M. Hammers, Achim J. Kaasch, Burkhart Schraven, Anne Dudeck, Sascha Kahlfuss

**Affiliations:** ^1^ Institute of Molecular and Clinical Immunology, Medical Faculty, Otto-von-Guericke University Magdeburg, Magdeburg, Germany; ^2^ Department of Dermatology, University of Luebeck, Luebeck, Germany; ^3^ Institute of Medical Microbiology and Hospital Hygiene, Medical Faculty, Otto-von-Guericke University Magdeburg, Magdeburg, Germany; ^4^ Health Campus Immunology, Infectiology and Inflammation (GCI-3), Medical Faculty, Otto-von-Guericke University Magdeburg, Magdeburg, Germany

**Keywords:** Th2 cells, tumor microenvironment, metabolism, tissue adaption, immune surveillance, immunometabolism

## Abstract

The function of T cells is critically dependent on their ability to generate metabolic building blocks to fulfil energy demands for proliferation and consecutive differentiation into various T helper (Th) cells. Th cells then have to adapt their metabolism to specific microenvironments within different organs during physiological and pathological immune responses. In this context, Th2 cells mediate immunity to parasites and are involved in the pathogenesis of allergic diseases including asthma, while CD8^+^ T cells and Th1 cells mediate immunity to viruses and tumors. Importantly, recent studies have investigated the metabolism of Th2 cells in more detail, while others have studied the influence of Th2 cell-mediated type 2 immunity on the tumor microenvironment (TME) and on tumor progression. We here review recent findings on the metabolism of Th2 cells and discuss how Th2 cells contribute to antitumor immunity. Combining the evidence from both types of studies, we provide here for the first time a perspective on how the energy metabolism of Th2 cells and the TME interact. Finally, we elaborate how a more detailed understanding of the unique metabolic interdependency between Th2 cells and the TME could reveal novel avenues for the development of immunotherapies in treating cancer.

## Introduction

Immune cells including T cells are capable of mounting immune responses against tumors. The ability of the immune system to detect and eliminate neoplastic cells is known as tumor immune surveillance (1, 2). Tumor immune surveillance is to a significant part mediated by CD8^+^ cytotoxic T lymphocytes (CTLs). During antitumor immune responses, CTLs recognize tumor peptides presented by major histocompatibility complexes (MHC) I through their T cell receptor (TCR). Consecutively, CTLs are capable to kill tumor cells by the release of perforins and granzyme B or by a mechanism that involves Fas ligand (FasL, CD95L)-mediated apoptosis of target cells (1, 2).

It is of note that also various CD4^+^ T cell populations have been detected in tumors, although their role within the tumor microenvironment (TME) is less well understood compared to CD8^+^ CTLs. For instance, CD4^+^ regulatory T cells (Tregs) are known to inhibit CTL function in tumors, while T helper (Th)1 cells participate in antitumor immunity by releasing IFN-γ and TNF (3–5). However, the role of Th2 cells and type 2 immunity during antitumor immune responses is less well understood. Th2 cells are best known to provide immunity against parasites and their pathogenic role in allergic diseases is well established and has been recently reviewed (6–8), while the regulation and function of Th2 cells in the TME is largely neglected and more controversial (9). Yet, therapeutically effective CD4^+^ CAR T cells have been shown to express a Th1 and, importantly, also a Th2 gene signature and to secrete both Th1 and Th2 cytokines (5, 10).

The TME is a unique metabolic niche, which differs between various tumor types and affected organs (11, 12). Importantly, many recently reviewed studies have shown that the TME and its metabolite composition directly affects the energy metabolism, gene expression and effector function of CTLs, Tregs and Th1 cells (11, 13, 14). How Th2 cells adapt to the TME and how tumors, vice versa, affect Th2 cell function is less established. In this review, we thus highlight studies investigating the metabolism of Th2 cells and summarize the role of Th2 cells in the TME. We then combine the evidence from both types of studies to discuss interactions of the TME and Th2 cell metabolism and function with a perspective and outline for future studies. At the end, we provide an outlook how a better understanding of this unique interdependency could help to establish new therapeutic strategies for cancer treatment.

### The Energy Metabolism of Th2 Cells

The type of energy metabolism that is used by T cells depends on their activation level, their differentiation phenotype and the specific environment they operate in (15–18). While naïve T cells primarily use oxidative lipid metabolism, their activation requires the supply with sufficient building blocks to guarantee proliferation. T cells fulfil these energy demands by metabolic reprogramming, which involves the upregulation of glycolysis and lipid metabolism (19, 20). High glycolysis rates require the activation of mammalian Target of Rapamycin (mTOR)1 and 2, Myc, Hypoxia-inducible factor 1α (HIF-1α) and increased expression of the glucose transporter (GLUT)1 (21–23). Lipid synthesis is promoted by the transcription factors mTORC1 and sterol regulatory element-binding protein (SREBP), which regulate critical enzymes involved in lipid metabolism such as the Acetyl-CoA carboxylase (ACC1) (24). Strikingly, pharmacological and genetic deletion of ACC1 completely prevents the generation of almost all effector Th cell lineages (25, 26).

Following activation in secondary lymphatic organs, T cells migrate to their target organs. The specific metabolic environment in these organs also affects the energy metabolism and effector function of different T cell populations, such as shown for instance in the melanoma TME, in which low glucose levels inhibit aerobic glycolysis and the tumoricidial function of CD4^+^ and CD8^+^T cells (27, 28). In this context, lung Th2 cells are mainly characterized by an upregulation of genes related to lipid oxidation and synthesis (29–31). **Table 1** summarizes studies investigating the Th2 cell metabolism in more detail (**Table 1**). Th2 cells in the lung were shown to upregulate the expression of the peroxisome proliferator-activated receptor-γ (PPAR-γ) (31, 43), a nuclear receptor that is induced by IL-4 receptor (IL-4R) ligation and signal transducer and activator of transcription (STAT6) activation (34, 44, 45). In lung Th2 cells, the transcription factor PPAR-γ regulates the expression of different genes that are critical for Th2 cell differentiation and effector function. Among them were the genes *Gata3*, *Stat5*, *Il5* and *Il13* (30, 39). In line with that observation, deletion of PPAR-γ in CD4^+^ T cells improved asthmatic airway inflammation and, on the flipside, reduced Th2-mediated immunity to *Heligmosomoides (H.) polygyrus* infection. In these studies, Th2 cells showed reduced IL-5 and IL-13 production in the absence of PPAR-γ (31, 43).

**Table 1:** Studies investigating the metabolism of Th2 cells.

18 (Review)	T cell metabolism drives T cell activation; T cell differentiation is linked to the environment via mTOR and AMPK
32 (Study)	PPAR-γ in CD4^+^ T cells induces genes involved in lipid metabolism
25 (Study)	Inhibition of ACC1 impairs differentiation and effector function of Th17 cells and other effector T cells
33 (Study)	mTOR signaling is responsive to hypoxia and therefor to metabolic cues
16, 17 (Review)	T cells metabolically adapt to specific microenvironments; Metabolism shapes T cell function and differentiation
31 (Study)	PPAR-γ is required for Th2 cytokine production
34 (Study)	PPAR-γ can be activated ligand independently to facilitate STAT6 signalling in macrophages
35 (Study)	Th1 and Th17 differentiation is dependent on mTORC1 and Th2 differentiation on mTORC2
15 (Review)	T cell metabolism is connected to T cell function, activation and differentiation
36	mTORC2 signaling is regulated by acetylation of Rictor. This might pose a metabolic sensor, as acetylation is acetyl-CoA dependent
37 (Study)	High intracellular AMP activates AMPK which phosphorylates Raptor and thereby inhibits mTORC1 signaling
38 (Study)	SGK1, a downstream target of mTORC2, regulates Th2, but also Th1 differentiation
39 (Review)	PPAR-γ controls Th2 cell-specific genes
40 (Study)	mTORC2 affects Th2, but also Th1 differentiation by distinct signaling pathways
26 (Study)	Deletion of ACC1 compromises CD8^+^ T cell differentiation
20 (Review)	Fatty acid synthesis is important for effector T cells; Fatty acid oxidation is critical for CD8^+^ T cells and Tregs
23 (Review)	GLUT1 and GLUT3 are important to fulfill the metabolic demands of effector CD4^+^ T Cells
41 (Study)	Glucose dependent acetylation of Rictor in GBM poses a metabolic sensor in mTORC2 signaling
19 (Study)	Upon activation T cells engage in anaerobic glycolysis via the upregulation of PDHK1; Cytokine synthesis is dependent on aerobic glycolysis
42 (Study)	mTORC2 signaling is responsive to ammonium
27 (Review)	T cell metabolism is shaped by internal and external environmental cues
43 (Study)	PPAR-γ exerts type 2 immunity in helminth infection but also in asthmatic airway inflammation
44 (Study)	PPAR-γ is required for alternative activation of macrophages
22 (Study)	Antitumor function of CD8^+^ T cells is dependent on HIF-1α-induced glycolysis
24 (Study)	mTORC1 induces lipid synthesis via SREBP and fatty acid synthesis enzyme transcription (e.g. ACC1)
29 (Review)	Th2 cells have characteristic metabolic profiles, which change dependent on maturation and location; PPAR-γ is an important transcription factor regulating the metabolism of Th2 cells in the lung
45 (Study)	PPAR-γ activation by IL-4 is mediated by STAT6 in dendritic cells and macrophages
30 (Study)	During asthmatic airway inflammation Th2 cells upregulate genes associated with lipid metabolism
46 (Review)	mTOR is a regulator of both, T cell differentiation and metabolism
21 (Study)	Myc induces glycolyisis upon t cell activation and links glutaminolysis to the synthesis of biosynthetic precursors
47 (Study)	mTORC1 is also important for Th2 differentiation

mTOR, mammalian Target of Rapamycin; AMPK, Adenosine monophosphate-activated protein kinase, PPAR-γ, Peroxisome proliferator-activated receptor γ, ACC, Acetyl-CoA carboxylase; IL, Interleukin; STAT, Signal transducer and activator of transcription; Treg, Regulatory t cell; GLUT, Glucose transporter; PDHK, Pyruvate dehydrogenase kinase; HIF, Hypoxia-inducible factor; SREBP, Sterol regulatory element-binding protein.

Importantly, in Th2 cells PPAR-γ also controls genes involved in fatty acid uptake and lipolysis (e.g. *Ldlr*, *Scrab2*, *Vdlr*, *Plin2*, and *Fabp5*). In addition, also pharmacological inhibition of glycolysis by 2-DG reduced Th2 cytokine production during asthmatic airway inflammation *in vivo* (30). A more detailed review of these mechanisms and the metabolic requirements of Th2 cells was recently published by Coquet and colleagues (29).

Taken together, early T cells undergo metabolic reprogramming and upregulate glycolysis to generate building blocks for proliferation. Following differentiation into the Th2 lineage, signals from inflamed tissues such as the lung during allergic asthma or parasite infection activate genes related to fatty acid uptake and lipid oxidation in Th2 cells.

### Th2 Cell-Mediated Immunity to Tumors

Compared to studies on CTLs, evidence for the function of Th2 cells in the TME is relatively spare. However, studies have shown that Th2 cells and type 2 immunity indeed take part in tumor immune surveillance by e.g. reducing the size of even established tumors (9, 48, 49; **Table 2**).

**Table 2:** Studies on the role of Th2 cells on tumors.

Evidence of Th2 cell antitumor activity	
50 (Study)	Perilymphatic injecton of IL-4 into CE-2 and TS/A tumors inhibits tumor growth and induces immune memory
48 (Review)	Th2 cells can inhibit but also facilitate tumor growth, progression and metastasis
51 (Study)	Th1 and Th2 cells are, unlike CD8^+^ T cells, independent of MHC I expression in tumor cells and play a significant role in initiating antitumor responses; Th2 cells exert antitumor effects via the recruitment of eosinophils
52, 53 (Study)	Eosinophil infiltration in oesophageal squamous cell carcinoma is a predictor of a favourable clinical outcome in humans
54 (Study)	Eosinophilic infiltration predicts survival in patients with gastric cancer
55 (Study)	Depletion of TGF-β receptor 2 disinhibits type 2 immunity to cancer in a breast cancer model by inducing tumor hypoxia and cell death through vasculature remodeling
56 (Study)	Transfer of Th2 cells eradicates subcutaneous myeloma and B cell lymphoma and is dependent on arginase produced by M2 macrophages
49 (Study)	Transfer of OVA-specific Th2 (but not Th1 cells) into tumor bearing mice cleared lung and visceral melanoma metastases via the recruitment of M2 macrophages
57 (Study)	Injection of IL-4 into mice prohibited TS/A tumor growth via the recruitment of eosinophils, neutrophils and macrophages into the TME
58 (Study)	TS/A tumors engineered to express IL-4 in mice were rejected via induction of necrotic areas by eosinophils and neutrophils
59, 60 (Study)	Eosinophilic infiltration predicts a favourable prognosis in patients with colorectal cancer
61 (Study)	Transfer of OVA-specific Th1 and Th2 cells in OVA expressing lymphomas is able to clear the tumors
62 (Study)	TS/A in mice engineered to express IL-4 were rejected in an eosinophil and CD8^+^ dependent way
63 (Review)^64^ (Study)	Eosinophiles are important in regulating immune responses in the TME and can exert anti-tumor effects in colorectal cancer
9 (Review)	Th2 cells can exert antitumor effects by recruiting eosinophils
65 (Study)	IL-4 exerts anti-tumor effects via the recruitment of eosinophils
66 (Study)	Enhanced IL-5 expression in mice protected from MCA-induced fibrosarcoma via the recruitment of eosinophils
67 (Study)	A type 2 immune microenvironment induced by a biologic urinary bladder matrix scaffold inhibits melanoma tumor function
**Evidence of Th2 cell pro tumor activity**	
68 (Study)	Breast cancer cells indirectly stimulate their own growth by instructing CD4^+^ T cells to secrete the Th2 cytokine IL-13
69 (Study)	IL-4 induces EMT in colon cancer cells via STAT6 dependent transcription of EMT promoting proteins
70 (Study)	IL-4 promotes the expression of antiapoptotic genes in various human cancers *in vitro*
71, 72 (Study)	Successful therapy of lung tumors and melanoma in mice was associated with a shift towards a Th1-Response
73 (Study)	Survival of patients with pancreatic cancer is negatively associated with Th2 cell infiltration
74 (Study)	IL-4 expressing CD4^+^ T cells enhance metastasis by instructing macrophages to activate epidermal growth factor signalling in breast cancer cells
75 (Study)	IL-5 in the tumor intestitial fluid is associated with a poor prognosis
76 (Study)	High risk HPV infections cause a type 2 inflammation and create an immunosuppressive environment
77 (Study)	IL-13 deficient mice showed a slower progression of prostate cancer
78 (Study)	IL-4 stimulates proliferation in human pancreatic cancer cells via MAPK, Akt-1, STAT3 and insulin receptor phosphorylation
79 (Review)	IL-4 and IL-13 receptors are possible targets for cancer therapy
80 (Review)	IL-13 negatively regulates antitumor immune surveillance
81 (Study)	Expression of the IL-4 receptor subunit α enhances malignancy of human pancreatic cancers *in vitro* and *in vivo*
82 (Study)	Th2 cell infiltration in human breast cancer is associated with unfavourable genetic properties of the tumour
83 (Study)	IL-5 facilitates lung metastasis from melanoma, lung and colon cancers via recruitment of eosinophils to the lung
84 (Study)	CCL5 recruits Th2 cells and mediates metastasis in breast cancer

IL, Interleukin; CE-2, Chemically induced fibrosarcoma; TS/A, Spontaneous adenocarcinoma; MHC, Major histocompatibility complex; CTL, Cytotoxic T lymphocyte; TGF, Tissue growth factor; OVA, Ovalbumin; TMA, Tumor microenvironment; MCA, Methylcholanthrene; EMT, Epithelial-mesenchymal transition; STAT, Signal transducer and activator of transcription; HPV, Human papillomavirus; MAPK, Mitogen activated protein kinase; CCL, chemokine C-C motif ligand.

A major difference between recognition of tumor cells by CD4^+^ T cells and CD8^+^ CTLs cells is that the latter detect antigens presented by MHC I complexes. However, tumors have the ability to develop mechanisms to evade recognition by CTLs (85–87). These mechanisms include the downregulation of MHC I molecules and/or losing the immunogenic target antigen CTLs are directed against (88). On the other side, tumor antigens can also be presented by bystander antigen presenting cells (APCs) *via* MHC II complexes to CD4^+^ T cells in tumors.

Through the MHC II complex pathway, Th2 cells can initiate antitumor responses. The involvement of type 2 immunity in antitumor immune responses is reflected by studies showing that IFN-γ-deficient and, importantly, also mice deficient for the Th2 cytokines IL-4 and IL-5 show reduced tumor clearance (51). Furthermore, injection of IL-4 enhanced tumor clearance and correlated with increased infiltration of eosinophils, macrophages, neutrophils and in part lymphocytes. In addition, neutralizing IL-5 by monoclonal antibodies restored tumor growth (50, 57, 58, 62, 65). The latter studies demonstrated that Th2 cytokines are important in anti-tumor immunity, although they do not provide direct evidence for an involvement of Th2 cells. With regard to this, it needs to be emphasized that Th2 cytokine production and type 2 immunity is not only mediated by Th2 cells but also to a significant part by type 2 innate lymphoid cells (ILC2s). ILC2s also secrete Th2 cytokines, depend on the Th2 transcription factor GATA3 but lack TCR expression (89–91). Currently, there is strong evidence that ILC2s contribute to anti-tumor immunity towards different types of tumors (92–94). However, ILC2s were also reported to be capable of exerting pro-tumor functions, revealing a more ambiguous role in immune responses against tumors than initially expected. The role of ILC2 in anti-tumor immunity was reviewed in-depth recently (95).

While the studies mentioned above report an involvement of type 2 immunity in anti-tumor immune responses, there are also further studies existing that show a direct influence of Th2 cells on tumor growth and progression: With regard to this, A20-Ovalbumin (OVA) expressing B cell lymphomas were cleared by the injection of either OVA-specific Th1 or, importantly, Th2 CD4^+^ T cells into tumor bearing host mice (61). Of note, another studied found that adoptively transferred OVA-specific CD4^+^ Th2 cells, but not Th1 cells, inhibit the growth of lung metastases produced by an OVA-transfected B16 melanoma (B16-OVA) (49). Importantly, the Th2-mediated antitumor response in the latter study was mediated through eosinophils and the expression of the eosinophil chemokine eotaxin. Confirmingly, eradication of tumors by adoptive transfer of tumor-specific Th2 cells was observed by another study (56). In addition, in a very recent study, Ming and colleagues showed that the transforming growth factor-β receptor 2 in CD4^+^ (but strikingly not CD8^+^) T cells is important in providing a host-directed protective Th2 response dependent on the Th2 cytokine IL-4 against tumors (96). The latter study provides strong and recent evidence that type 2 immunity mediates anti-tumor effects through tissue defense mechanisms.

Immunity to tumors by Th2 cells is to a significant part mediated by Th2 cytokines and through secondary recruitment of tumoricidal myeloid cells such as eosinophils, which often act in concert with macrophages (49, 67). Indeed, depletion of granulocytes completely abolished anti-tumor immunity (48, 62). Furthermore, IL-5-deficicent mice showed impaired numbers of eosinophils in the tumor and a consecutive loss of anti-tumor immunity (51). Conversely, IL-5 overexpressing mice develop fewer tumors and, if so, had high eosinophil numbers within the TME (66). In line with this observation, others reported an association of increased eosinophil numbers and an overall prolonged survival (52–54, 59, 60). Besides eosinophils and alternative activated M2 macrophages, also mast cells, B cells and type 2 CD8^+^ T cells contribute to Th2-mediated anti-tumor immunity, which was, however, reviewed elsewhere (48). In this context, it needs to be emphasized that especially mast cells are also strong producers of Th2 cytokines and can exert pro- and anti-tumor effects (97). So far, however, strongest evidence comes from studies demonstrating anti-tumor activity of eosinophils (63, 64).

Of note, there is also evidence suggesting that Th2 immunity promotes cancer genesis, progression and metastasis. One study for instance found that remission of transplanted lung tumors into mice was accompanied by a shift from Th2 towards Th1 cells. In this study, the authors found that a predominant Th2 response in the TME is associated with an unfavorable outcome (71). One report showed a Th1 skewing to be associated with successful immune modulatory therapy of cancer (72). Others demonstrated an association of Th2 cells in the TME with the progression of breast cancer and cervical neoplasia (75, 76, 82). In addition, in breast cancer, colorectal cancer and lung cancer, type 2 immunity has been shown to enhance metastasis (69, 74, 83, 84). As possible mechanisms for the above listed observations, direct effects of IL-4 on cancer cells, an increase in tumor-associated macrophages and IL-5-dependent eosinophil recruitment at the site of metastasis were discussed. In general, many studies suggest a direct effect of Th2 cytokines on cancer cells and tumor progression, while others lack mechanistic explanations. Of note, direct evidence for pro-tumor effects of Th2 cells, particularly, does not exist (68, 70, 77, 78, 80, 81).

Taken together, Th2 cells were shown to mediate pro- and anti-tumor effects. While, traditionally, type 2 immunity was implicated in an inhibition of anti-tumor responses, a number of studies provide convincing evidence demonstrating that Th2 cells indeed mediate anti-tumor immunity. Of note, these studies do not only provide correlative analyses, but apply adoptive Th2 cell transfer experiments (49, 56, 61) or perform pharmacological administration of Th2 cytokines to tumor bearing mice (50, 57, 58, 62, 66). Some of the anti-tumor effects of Th2 cells in addition were attributed to an indirect effect of Th2 cells and their cytokines on Th9 cells (98, 99).

In summary, whether Th2 cells and type 2 immunity exert pro- or anti-tumor effects seems to be strongly depending on the type and stage of tumor (context dependency). However, in this review we focus on the anti-tumor role of Th2 cells and type 2 immunity as most of the mechanistic and experimental (and not only correlative) data derives from studies, which postulate an anti-tumor function of Th2 cells.

### The Tumor Microenvironment (TME)

Tumor cells are recognized and eliminated by innate and adaptive immune cells. Thus, the immune system in principle has an impressive ability to keep neoplastic cells in check before tumor cells enter uncontrolled expansion (1, 2). The fact that the immune system can attack tumors is the basis for antitumor immunotherapy such as immune checkpoint blockade or adoptive cell transfer of engineered T cells (100, 101). However, tumors possess several mechanisms to evade tumor immune surveillance (102), which is why some patients do not respond to immunotherapy. To a significant part, these evasion mechanisms involve the metabolic modulation of the TME (11, 103, 104). The metabolic TME is mainly characterized by a high cancer cell metabolism and a consecutive starvation of immune cells including effector T cells.

Tumors enable their rapid growth rate through glycolysis, which generates metabolic intermediates for the synthesis of amino acids, nucleotides, and fatty acids (105). Through glycolysis, tumor cells consume high levels of glucose from their surrounding environment and produce high amounts of lactate (106, 107). Both, reduced glucose and high lactate concentrations within the TME, results in immunosuppression (108, 109). Differentiation of naïve T cells into effector T cells crucially depends on metabolic reprogramming, which is facilitated by glucose uptake through GLUT1 and aerobic glycolysis (19, 21–23). Glucose deprivation in the TME thus cumulates in effector T cell hyporesponsiveness. Mechanistically, low glucose levels in the TME reduce AKT activity and induce apoptosis in CTLs through the activation of proapoptotic B-cell lymphoma-2 (Bcl-2) family members (110, 111). The latter mechanism likely also applies to various CD4^+^ Th cell populations in the TME, although direct evidence is missing so far. Reduced glucose levels also decrease the levels of the intermediate phosphoenolpyruvate in T cells, which impairs calcium flux and nuclear factor of activated T cells (NFAT) signaling (112). In addition, high lactate in the TME further impairs NFAT activation and its translocation to the nucleus (113). As calcium-mediated NFAT signals control metabolic reprogramming of T cells by regulating gene expression of several glycolytic enzymes (114), T cells in the TME show impaired activation, proliferation and effector function against neoplastic cells. Importantly, acidification of the TME through high lactate concentrations impairs effector T cell function stronger than the function of Tregs. This likely occurs as Tregs also use fatty acid oxidation and might even use lactate as fuel (115–117). In view of the fact that also tissue Th2 cells seem to use lipid metabolism preferentially, it is tempting to speculate that also Th2 cells could be more resistant to high lactate concentrations and a low pH within the TME.

In addition to low glucose concentrations within tumors, the TME is also depleted of specific amino acids including glutamine, alanine, tryptophan, arginine, cysteine and ornithine, some of which were shown to be important for T cell proliferation and effector function (118–120). Importantly, upon T cell activation several genes e.g. those encoding for amino acid transporters are upregulated (121), which indicates a high demand for the exchange of amino acids for clonal expansion of T cells following antigen encounter.

While many studies have focused on glucose and amino acid metabolism, less studies report on lipid metabolism in the TME. Generally, lipids play a significant role in cancer progression (122, 123). Cancer cells are capable of inducing lipolysis in adjacent adipocytes and fatty acid synthesis in cancer associated fibroblasts. This has been demonstrated for melanoma (124, 125), breast cancer (126, 127), ovarian cancer (128), prostate cancer (129) and pancreatic cancer (130).

Another characteristic of especially necrotic tumors is that the TME consist of a specific ion composition that is characterized by high potassium levels due to necrotic cell lysis (131). Increased potassium concentrations in the extracellular fluid of mouse and human tumors suppress CD8^+^ CTL function, while overexpression of the voltage-gated potassium channel K_v_1.3 (that transports potassium outside the cell) improved CTL effector function and survival of melanoma bearing mice (131). Importantly, preventing calcium influx through genetic deletion of components of the calcium release-activated calcium channel (CRAC) pathway impairs CD8^+^ CTL effector function and antitumor immunity in mouse models for melanoma and colon carcinoma (132). In addition to calcium also sodium was shown to regulate T cell function, more precisely, the effector function of Th17 cells (133–136). However, the role of sodium on effector T cell function within the TME is elusive. The above studies (131, 132) show that ions have significant impact on effector T cell function against tumors. However, despite these studies the impact of ions on adaptive and innate immune cells in the TME but also in other tissues is still largely neglected and not well understood.

Hypoxia is another prominent feature of the TME (137). The high metabolic rate of tumor cells in combination with an insufficient vascularization especially of large solid tumors leads to a TME that is characterized by reduced oxygen levels. Hypoxia within the TME was on the one hand reported to increase CTL-mediated tumor cell killing through increasing the packaging of granzyme B. This was associated with prolonged survival in the B16-OVA melanoma mouse model (138). In contrast, others have shown that T cells in fact avoid areas of hypoxia and have reported an immunosuppressive function of hypoxia on effector T cells in the TME (109, 139, 140). In that context, the oxygen-sensing prolyl-hydroxylase (PHD) was reported to limit Th1 and CTL function, while PHD promoted Treg differentiation following tumor colonization in the lung (141). HIF-1α, an important transcription factor in sensing oxygen levels, was shown to positively control PD-L1 expression in myeloid derived suppressor cells (MDSCs). This induced T cell exhaustion but promoted generation of Tregs (142, 143). Of note, deletion of HIF-1α increased fatty acid catabolism (oxidation) and improves PPAR-α signaling in CD8^+^ CTLs (144). As HIF-1α negatively regulates fatty acid oxidation and tissue Th2 cells predominantly use lipid metabolism, this could have important mechanistic implications how hypoxic TME regulate Th2 cell metabolism and function.

In addition to the above discussed evasion mechanisms, neoplastic cells also produce several metabolic intermediates, which affect the functions of various immune cells including T cells within the TME. In malignant cells Indoleamine 2,3-dioxygenase (IDO) is upregulated (145). IDO degrades tryptophan to kynurenine, which inhibits effector T cells and promotes Treg differentiation within tumors (104). Importantly, mass spectroscopy of tumor fluid from tumor bearing mice has already in part allowed characterizing metabolite composition within the TME of a handful of tumors (107).

## Discussion

### Interactions of Th2 Cell Metabolism, TME, and Potential Therapeutic Strategies

Current evidence indicates that Th2 cell-mediated type 2 immune responses can contribute to anti-tumor immunity, although for some tumors Th2 cells and/or cytokines have also been shown to promote tumor growth and metastasis.

**Figure 1:**
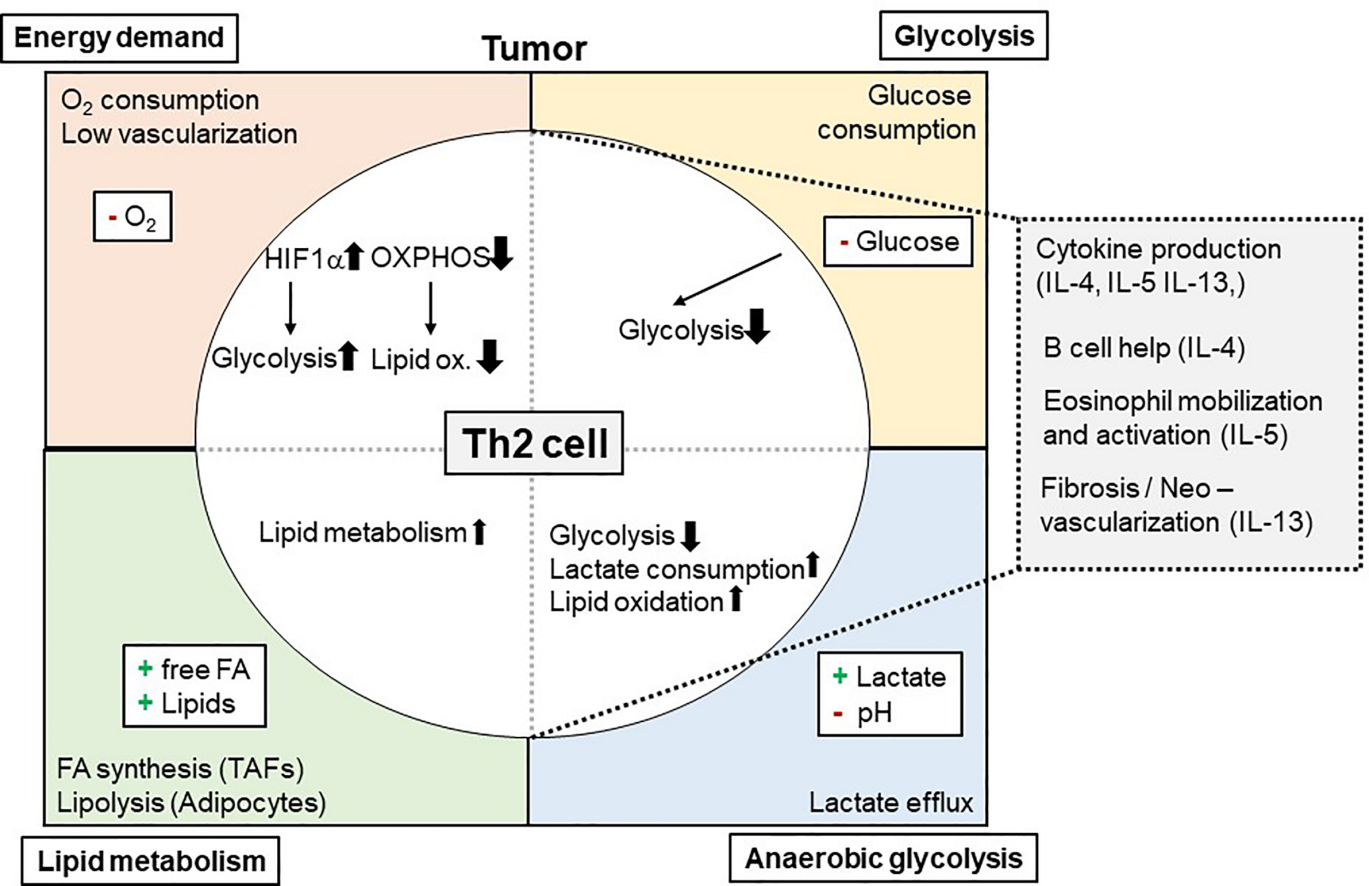
Metabolic Interdependency of Th2 cell-mediated type 2 immunity and the Tumor Microenvironment (TME): The TME is characterized by a high energy demand, glycolysis, and lipid metabolism. This reduces 0_2_ and glucose availability within the TME, while free FA, other lipids and lactate is found to be elevated. These conditions affect Th2 cells and their effector functions (e.g. cytokine production of IL-4, IL-5, and IL-13) and, secondary, eosinophil mobilization and activation. 0_2_, Oxygen; IL, Interleukin; Ox., Oxidation; FA, Fatty acid(s).

One way how tumors can influence Th2 cell-mediated immunity to neoplastic cells is by consuming nutrients and thereby starving Th2 effectors. This mechanism was meanwhile indicated for several immune cells within the TME including macrophages, CD8^+^ T cells, dendritic cells (DCs), and natural killer cells but not yet for Th2 cells (130, 146–149). Tumors consume high levels of glucose to perform glycolysis. Glucose is on the other hand essential for metabolic reprogramming of naïve T cells when becoming effector T cells (19–23). While tissue Th2 cells in the allergic lung were dependent on lipid metabolism, also pharmacological inhibition of glycolysis by the glucose analogue 2-DG attenuated asthmatic airway inflammation by interfering with Th2 cytokine production (30). This indicates that also in the TME Th2 cells likely could be affected by low glucose levels (**Figure 1**). However, direct evidence whether and how reduced glucose concentrations within the TME affect Th2 cells is so far elusive.

In recent literature, a Th2 cell-specific utilization of lipid metabolism pathways has been described (29–31, 150). In this context, tissue Th2 cell activation was shown to involve PPAR-γ activation (31, 43). PPAR-γ, in turn, controlled Th2 cell gene expression and the expression of genes associated with lipid metabolism (29, 30, 39). Of note, Coquet and colleagues reported reduced T cell numbers in the bronchoalveolar lavage following house dust mite-induced asthmatic airway inflammation after *in vivo* administration of orlistat or etomoxir, which block fatty acid synthesis and uptake or fatty acid oxidation, respectively (30). It is of note, that these drugs also inhibited ILC2s, which resulted in elevated helminth burden after infection with *Trichuris muris* (151). This dependency on lipid metabolism could mean a significant advantage for Th2 cells (and ILC2) in the TME for at least two reasons: First, availability of lipids within the TME might be even higher than in healthy tissues. In addition, as Th2 cells are capable of utilizing lipids in the TME, this could maintain their function even when being exposed to low glucose environments (**Figure 1**).

Given this evidence, therapy regimens that involve the modulation of the Th2 cell metabolism are thinkable. One potential molecular target could be indeed the transcription factor PPAR-γ as it facilitates lipid metabolism of Th2 cells. Thiazolidinediones (TZDs), a group of diabetes drugs that activate PPAR-γ, have already been shown to be associated with a lower risk for cancer (152, 153). While this might be mediated by the antidiabetic effects of TZDs, there is also evidence for TZDs directly inhibiting growth of established tumors. Moreover, TZDs are in fact discussed for cancer therapy (154, 155). One concern using TZDs is, however, the notion that PPAR-γ also plays an important role in Tregs as Treg accumulation in adipose tissue has been shown to be PPAR-γ-dependent (156). If this mechanism also applies to the TME, this could lead to a net pro-tumor effect of TZDs.

Apart from targeting only the metabolism of immune cells, it appears also plausible to target the metabolism of tumor cells simultaneously. With regard to this, inhibition of cancer-specific metabolic pathways in cancer therapy has been extensively discussed in recent years (109, 157, 158). First, disturbing tumor metabolism might abrogate tumor growth directly. On the other hand, a consecutive secondary change in the metabolic TME might further improve immune cell function. Of note, a major challenge of this approach is the inhibition of tumor metabolism without compromising essential metabolic pathways in T cells at the same time (159). It is tempting to speculate that Th2 cell-mediated anti-tumor responses could be enhanced by higher glucose and/or oxygen levels as Th2 cells have been shown to be impaired by glycolysis inhibition (30) and because oxygen is essential for lipid metabolism predominantly performed by Th2 cells (29).

Of note, especially large tumors consist of several hypoxic areas. This might hinder Th2 cell mediated anti-tumor immunity for two reasons (**Figure 1**): First, lipid oxidation is oxygen dependent. Therefore, it still needs to be elucidated whether lipid oxidation, as proposed above, could pose a salvage pathway for Th2 cells in the TME. Yet, hypoxia was reported to induce a Th2-skewing phenotype of DCs, which could promote anti-tumor immunity (160, 161). Secondly, hypoxia normally increases the expression and function of the transcription factor HIF-1α. HIF-1α is on the one hand a positive regulator of glycolysis but also negatively regulates lipid metabolism (162–164). Thus, increased HIF-1α expression in Th2 cells within hypoxic tumor areas could prevent the usage of lipid metabolism by Th2 cells as an alternative energy pathway when glucose is missing. On the other hand, HIF-1α inhibition could serve as strategy to improve Th2-cell mediated immunity to tumors. Although upregulation of glycolysis was shown to be critical for initial T cell activation, and Myc together with HIF-1α enables metabolic reprogramming of T cells (19–21), it is not clear whether Th2 differentiation is dependent to the same extend on HIF-1α activation. So far, HIF-1α-mediated glycolysis was shown to be important mainly for Th17, but not for Th1 and Th2 differentiation (21, 165). Another important metabolic regulator upstream of HIF1-α is mTORC, which controls several key transcription factors including HIF-1α, Myc, PPARα, PPARγ and SREBP (46). In particular, mTORC2 has been suggested to selectively mediate Th2 polarization, whereas mTORC1 was indicated to be important in Th1 and Th17 differentiation (35, 38). Others, however, reported mTORC1 to be important for Th2- and mTORC2 for Th1 differentiation (40, 47). While mTORC1 has been shown to respond to various metabolic conditions like low energy supply and hypoxia (37, 41), mTORC2 has been shown to be regulated by metabolic cues such as glucose availability (41, 42). Moreover, mTORC2 is discussed to act as a sensor for Acetyl-CoA and NAD^+^ levels and therefore to detect the overall energy status of cells (36).

In principle, Th2 cells may be used for advanced adoptive cell transfer therapies. So far, CAR T cell therapies have been (often) unsuccessful for treatment of solid tumors as the latter possess several mechanisms to escape immune responses (166, 167). In addition, there are only a few studies so far investigating Th2 cell adoptive cell transfer to treat tumors in mice (49, 56, 61).

For tumor cells a high IDO expression was reported (145). Importantly, IDO is also expressed by innate immune cells such as DCs that take part in anti-tumor immunity (168). CD4^+^ T cells co-cultured with IDO-deficient lung DCs produced less Th2 cytokines. High IDO activity in tumors and DCs could thus secondarily amplify Th2 cell responses against tumors. However, this assumption is complicated by the fact that there also exists evidence that kynurenine metabolites can negatively regulate T cell function by inducing apoptosis (169). In addition, studies have shown that genetic or pharmacological deletion of IDO restores anti-tumor immunity (170). This is the case as IDO is involved in the generation of Tregs and MDSCs, which both suppress the function of CTLs and other effector cells within the TME (105, 171). In line with this observation studies reported that kynurenine induces the transcription factor aryl hydrocarbon receptor (AHR) that is important for differentiation of naïve T cells into Tregs (172, 173). As IDO inhibitors are currently in clinical trials for different cancer types including bladder cancer, endometrial cancer or head and neck squamous cell carcinoma (174) it would be of great interest to characterize the immune cell repertoire in the TME upon IDO inhibition.

Another tissue factor that influences T cell function in the TME is the ion composition and concentration. As calcium signaling plays a role in metabolic reprogramming of T cells, an effect of ions in the TME on T cell metabolism seems plausible. However, while there is clear and strong evidence that calcium and potassium regulates the function of CTLs (132, 175, 176), there is a gap in our understanding how ions influence the function of Th2 cells in tumors.

## Conclusions

To develop tailor-made cancer therapies that target specific immune cell populations such as Th2 cells by modulating their metabolism, a detailed understanding of the TME of various tumor subtypes is needed, and still some challenges have to be overcome. One important questions is whether Th2 cells, based on their metabolic profile, are able to function in the glucose and amino acid and oxygen depleted TME.

Despite great advances in the field, a lot of evidence for the metabolic regulation of various Th cells still is based on experiments using *in vitro* culture systems and artificial media. To overcome this, some strategies were recently discussed by Jones and colleagues (108). First, the design of physiologic media resembling the specific metabolite (and at best also ion-) composition of different compartments can help in elucidating the relationship of immune cells and their surrounding environment. Second, tumor spheroids and organoid models and the detailed characterization of the TME *in vivo* will help to understand the complex relationship and metabolic interdependency between tumors and immune cells better.

Furthermore, a detailed understanding of the TME of various tumor subtypes is needed to identify tumors with a Th2 advantageous TME. The decision whether Th2 cells and type 2 immunity provides pro- or anti-tumor immune responses is strongly dependent on the type and stage of the individual tumor. Considering the utilization of lipid metabolism pathways by Th2 cells and the capability of tumors to induce lipolysis, especially tumors with adjacent adipose tissue could be of significant advantage. Especially melanoma might be promising for two reasons: First, melanoma are close to a source of lipids, namely the subcutaneous adipose tissue and have been shown to induce lipolysis in adjacent adipocytes (124, 125). Second, adoptive cell therapy by using Th2 cells has already been shown to be effective in melanoma animal models (49).

In conclusion, the special metabolic characteristics of Th2 cells might prove advantageous when therapeutically modulating the TME to treat cancer. Especially Th2 cell utilization of lipids might be useful. In future, a precise characterization of the TME in different tumors, a specification of the role of lipid metabolism in tissue Th2 cells and a more direct observation of Th2 cells in the TME *in vivo* or during experiments with artificial environments and a defined nutrient composition *in vitro* are needed. Of note, in tumors where Th2 cells exert pro-tumor effects, an inhibition of metabolic pathways used by Th2 cells is thinkable, vice versa.

## Author Contributions

SS and SK drafted the manuscript. All authors contributed to the article and approved the submitted version.

## Conflict of Interest

The authors declare that the research was conducted in the absence of any commercial or financial relationships that could be construed as a potential conflict of interest.

## References

[1] Crispin JC Tsokos GC. Cancer Immunosurveillance by CD8 T Cells. F100 Faculty Rev (2020) 9:F1000. 10.12688/f1000research.21150.1 PMC7001753 32089832

[2] St Paul M Ohashi PS. The Roles of CD8 + T Cell Subsets in Antitumor Immunity. Trends Cell Biol (2020) 30(9):695–704. 10.1016/j.tcb.2020.06.003 32624246

[3] Facciabene A Motz GT Coukos G. T Regulatory Cells: Key Players in Tumor Immune Escape and Angiogenesis. Cancer Res (2012) 72(9):2162–71. 10.1158/0008-5472.CAN-11-3687 PMC3342842 22549946

[4] Magen A Nie J Ciucci T Tamoutounour S Zhao Y Mehta M. Single-Cell Profiling Defines Transcriptomic Signatures Specific to Tumor-Reactive Versus Virus-Responsive Cd4 + T Cells. Cell Rep (2019) 29(10):3019–32.e6. 10.1016/j.celrep.2019.10.131 31801070 PMC6934378

[5] Tay RE Richardson EK Toh HC. Revisiting the Role of CD4^+^ T Cells in Cancer Immunotherapy—New Insights Into Old Paradigms. Cancer Gene Ther (2021) 28:5–17. 10.1038/s41417-020-0183-x 32457487 PMC7886651

[6] Ruterbusch M Pruner KB Shehata L Pepper M. In Vivo Cd4 + T Cell Differentiation and Function: Revisiting the Th1/Th2 Paradigm. Annu Rev Immunol (2020) 38:705–25. 10.1146/annurev-immunol-103019-085803 32340571

[7] von Moltke J Pepper M. Sentinels of the Type 2 Immune Response. Trends Immunol (2018) 39(2):99–111. 10.1016/j.it.2017.10.004 29122456 PMC6181126

[8] Lambrecht BN Hammad H. The Immunology of Asthma. Nat Immunol (2015) 16(1):45–56. 10.1038/ni.3049 25521684

[9] Simson L Ellyard JI Parish CR. The Role of Th2-Mediated Anti-Tumor Immunity in Tumor Surveillance and Clearance. In: Penichet M Jensen-Jarolim E, editors. Cancer and Ige. Totowa, NJ: Humana Press (2010). 10.1007/978-1-60761-451-7_11

[10] Xhangolli I Dura B Lee G Kim D Xiao Y Fan R. Single-Cell Analysis of CAR-T Cell Activation Reveals a Mixed TH1/TH2 Response Independent of Differentiation. Genomics Proteom Bioinforma (2019) 17:129–39. 10.1016/j.gpb.2019.03.002 PMC6620429 31229590

[11] Lim AR Rathmell WK Rathmell JC. The Tumor Microenvironment as a Metabolic Barrier to Effector T Cells and Immunotherapy. eLife (2020) 9:e55185. 10.7554/eLife.55185 32367803 PMC7200151

[12] Reznik E Luna A Aksoy BA Liu EM La K Ostrovnaya I. A Landscape of Metabolic Variation Across Tumor Types. Cell Syst (2018) 6(3):301–3.e3. 10.1016/j.cels.2017.12.014 29396322 PMC5876114

[13] Wang H Franco F Ho P-C. Epub 2017 Jul 14. Metabolic Regulation of Tregs in Cancer: Opportunities for Immunotherapy. Trends Cancer (2017) 3(8):583–92. 10.1016/j.trecan.2017.06.005 28780935

[14] Guerra L Bonetti L Brenner D. Metabolic Modulation of Immunity: A New Concept in Cancer Immunotherapy. Cell Rep (2020) 32(1):107848. 10.1016/j.celrep.2020.107848 32640218

[15] Geltink RIK Kyle RL Pearce EL. Unraveling the Complex Interplay Between T Cell Metabolism and Function. Annu Rev Immunol (2018) 36:461–88. 10.1146/annurev-immunol-042617-053019 PMC6323527 29677474

[16] Buck MD O’Sullivan D Pearce EL. T Cell Metabolism Drives Immunity. J Exp Med (2015) 212(9):1345–60. 10.1084/jem.20151159 PMC4548052 26261266

[17] Buck MD Sowell RT Kaech SM Pearce EL. Metabolic Instruction of Immunity. Cell (2017) 169(4):570–86. 10.1016/j.cell.2017.04.004 PMC5648021 28475890

[18] Almeida L Lochner M Berod L Sparwasser T. Metabolic Pathways in T Cell Activation and Lineage Differentiation. Semin Immunol (2016) 28(5):514–24. 10.1016/j.smim.2016.10.009 27825556

[19] Menk AV Scharping NE Moreci RS Zeng X Guy C Salvatore S. Early Tcr Signaling Induces Rapid Aerobic Glycolysis Enabling Distinct Acute T Cell Effector Functions. Cell Rep (2018) 22(6):1509–21. 10.1016/j.celrep.2018.01.040 PMC5973810 29425506

[20] Lochner M Berod L Sparwasser T. Fatty Acid Metabolism in the Regulation of T Cell Function. Trends Immunol (2015) 36:81–91. 10.1016/j.it.2014.12.005 25592731

[21] Wang R Dillon CP Shi LZ Milasta S Carter R Finkelstein D. Pmcid: Pmc3248798. The Transcription Factor Myc Controls Metabolic Reprogramming Upon T Lymphocyte Activation. Immunity (2011) 35(6):871–82. 10.1016/j.immuni.2011.09.021 PMC3248798 22195744

[22] Palazon A Tyrakis PA Macias D Veliça P Rundqvist H Fitzpatrick S. An HIF-1α/Vegf-a Axis in Cytotoxic T Cells Regulates Tumor Progression. Cancer Cell (2017) 32(5):669–683.e5. 10.1016/j.ccell.2017.10.003 29136509 PMC5691891

[23] Macintyre AN Gerriets VA Nichols AG Michalek RD Rudolph MC Deoliveira D. The Glucose Transporter Glut1 Is Selectively Essential for CD4 T Cell Activation and Effector Function. Cell Metab (2014) 20:61–72. 10.1016/j.cmet.2014.05.004 24930970 PMC4079750

[24] Porstmann T Santos CR Griffiths B Cully M Wu M Leevers S. SREBP Activity Is Regulated by Mtorc1 and Contributes to Akt-Dependent Cell Growth. Cell Metab (2008) 8:224–36. 10.1016/j.cmet.2008.07.007 PMC2593919 18762023

[25] Berod L Friedrich C Nandan A Freitag J Hagemann S Harmrolfs K. De Novo Fatty Acid Synthesis Controls the Fate Between Regulatory T and T Helper 17 Cells. Nat Med (2014) 20:1327. 10.1038/nm.3704 25282359

[26] Lee JE Walsh MC Hoehn KL James DE Wherry EJ Choi Y. Regulator of Fatty Acid Metabolism, Acetyl Coa Carboxylase 1 (ACC1), Controls T Cell Immunity. J Immunol (2014) 192(7):3190–9. 10.4049/jimmunol.1302985 PMC3965631 24567531

[27] Munford H Dimeloe S. Intrinsic and Extrinsic Determinants of T Cell Metabolism in Health and Disease. Front Mol Biosci (2019) 6:118. 10.3389/fmolb.2019.00118 31709265 PMC6823819

[28] Ho PC Bihuniak JD Macintyre AN Staron M Liu X Amezquita R. Phosphoenolpyruvate Is a Metabolic Checkpoint of Anti-Tumor T Cell Responses. Cell (2015) 162(6):1217–28. 10.1016/j.cell.2015.08.012 PMC4567953 26321681

[29] Stark JM Tibbitt CA Coquet JM. The Metabolic Requirements of Th2 Cell Differentiation. Front Immunol (2019) 10:2318. 10.3389/fimmu.2019.02318 31611881 PMC6776632

[30] Tibbitt CA Stark JM Martens L Ma J Mold JE Deswarte K. Single-Cell RNA Sequencing of the T Helper Cell Response to House Dust Mites Defines a Distinct Gene Expression Signature in Airway Th2 Cells. Immunity (2019) 51:1–16. 10.1016/j.immuni.2019.05.014 31231035

[31] Chen T Tibbitt CA Feng X Stark JM Rohrbeck L Rausch L. Ppar-γ Promotes Type 2 Immune Responses in Allergy and Nematode Infection. Sci Immunol (2017) 2(9):eaal5196. 10.1126/sciimmunol.aal5196 28783701

[32] Angela M Endo Y Asou HK Yamamoto T Tumes DJ Tokuyama H. Fatty Acid Metabolic Reprogramming Via Mtor-Mediated Inductions of Pparγ Directs Early Activation of T Cells. Nat Commun (2016) 7:13683. 10.1038/ncomms13683 27901044 PMC5141517

[33] Brugarolas J Lei K Hurley RL Manning BD Reiling JH Hafen E. Regulation of Mtor Function in Response to Hypoxia by REDD1 and the TSC1/TSC2 Tumor Suppressor Complex. Genes Dev (2004) 18(23):2893–904. 10.1101/gad.1256804 PMC534650 15545625

[34] Daniel B Nagy G Czimmerer Z Horvath A Hammers DW Cuaranta-Monroy I. The Nuclear Receptor Pparγ Controls Progressive Macrophage Polarization as a Ligand-Insensitive Epigenomic Ratchet of Transcriptional Memory. Immunity (2018) 49:615–26. 10.1016/j.immuni.2018.09.005 PMC6197058 30332629

[35] Delgoffe G Pollizzi K Waickman A Heikamp E Meyers DJ Horton MR. The Kinase Mtor Regulates the Differentiation of Helper T Cells Through the Selective Activation of Signaling by Mtorc1 and Mtorc2. Nat Immunol (2011) 12:295–303. 10.1038/ni.2005 21358638 PMC3077821

[36] Glidden EJ Gray LG Vemuru S Li D Harris TE Mayo MW. Multiple Site Acetylation of Rictor Stimulates Mammalian Target of Rapamycin Complex 2 (Mtorc2)-Dependent Phosphorylation of Akt Protein. J Biol Chem (2012) 287(1):581–8. 10.1074/jbc.M111.304337 PMC3249112 22084251

[37] Gwinn DM Shackelford DB Egan DF Mihaylova MM Mery A Vasquez DS. AMPK Phosphorylation of Raptor Mediates a Metabolic Checkpoint. Mol Cell (2008) 30(2):214–26. 10.1016/j.molcel.2008.03.003 PMC2674027 18439900

[38] Heikamp EB Patel CH Collins S Waickman A Oh MH Sun IH. the Agc Kinase Sgk1 Regulates Th1 and Th2 Differentiation Downstream of the Mtorc2 Complex. Nat Immunol (2014) 15:457–64. 10.1038/ni.2867 PMC4267697 24705297

[39] Henriksson J Chen X Gomes T Ullah U Meyer KB Miragaia R. Genome-Wide CRISPR Screens in T Helper Cells Reveal Pervasive Crosstalk Between Activation and Differentiation. Cell (2019) 176:882–96. 10.1016/j.cell.2018.11.044 PMC6370901 30639098

[40] Lee K Gudapati P Dragovic S Spencer C Joyce S Killeen N. Mammalian Target of Rapamycin Protein Complex 2 Regulates Differentiation of Th1 and Th2 Cell Subsets Via Distinct Signaling Pathways. Immunity (2010) 32(6):743–53. 10.1016/j.immuni.2010.06.002 PMC2911434 20620941

[41] Masui K Tanaka K Ikegami S Villa GR Yang H Yong WH. Glucose-Dependent Acetylation of Rictor Promotes Targeted Cancer Therapy Resistance. Proc Natl Acad Sci USA (2015) 112(30):9406–11. 10.1073/pnas.1511759112 PMC4522814 26170313

[42] Merhi A Delrée P Marini AM. The Metabolic Waste Ammonium Regulates Mtorc2 and Mtorc1 Signaling. Sci Rep (2017) 7:44602. 10.1038/srep44602 28303961 PMC5355986

[43] Nobs SP Natali S Pohlmeier L Okreglicka K Schneider C Kurrer M. Pparγ in Dendritic Cells and T Cells Drives Pathogenic Type-2 Effector Responses in Lung Inflammation. J Exp Med (2017) 214(10):3015–35. 10.1084/jem.20162069 PMC5626395 28798029

[44] Odegaard JI Ricardo-Gonzalez RR Goforth MH Morel CR Subramanian V Mukundan L. Macrophage-Specific Ppargamma Controls Alternative Activation and Improves Insulin Resistance. Nature (2007) 447:1116–20. 10.1038/nature05894 PMC2587297 17515919

[45] Szanto A BL B ZS N Barta E Dezso B Pap A. STAT6 Transcription Factor Is a Facilitator of the Nuclear Receptor Pparγ-Regulated Gene Expression in Macrophages and Dendritic Cells. Immunity (2010) 33:699–712. 10.1016/j.immuni.2010.11.009 21093321 PMC3052437

[46] Waickman AT Powell JD. Mtor, Metabolism, and the Regulation of T-Cell Differentiation and Function. Immunol Rev (2012) 249(1):43–58. 10.1111/j.1600-065X.2012.01152.x 22889214 PMC3419491

[47] Yang K Shrestha S Zeng H Karmaus PW Neale G Vogel P. T Cell Exit From Quiescence and Differentiation Into Th2 Cells Depend on Raptor-Mtorc1-Mediated Metabolic Reprogramming. Immunity (2013) 39(6):1043–56. 10.1016/j.immuni.2013.09.015 PMC3986063 24315998

[48] Ellyard JI Simson L Parish CR. Th2-Mediated Anti-Tumour Immunity: Friend or Foe? Tissue Antigens (2007) 70(1):1–11. 10.1111/j.1399-0039.2007.00869.x 17559575

[49] Mattes J Hulett M Xie W Hogan S Rothenberg ME Foster P. Immunotherapy of Cytotoxic T Cell-Resistant Tumors by T Helper 2 Cells: An Eotaxin and STAT6-Dependent Process. J Exp Med (2003) 197:387–93. 10.1084/jem.20021683 PMC2193835 12566422

[50] Bosco M Giovarelli M Forni M Modesti A Scarpa S Masuelli L. Low Doses of IL-4 Injected Perilymphatically in Tumor-Bearing Mice Inhibit the Growth of Poorly and Apparently Nonimmunogenic Tumors and Induce a Tumor-Specific Immune Memory. J Immunol (1990) 145:3136–43. 10.3109/08830189809084486 2212677

[51] Hung K Hayashi R Lafond-Walker A Lowenstein C Pardoll D Levitsky H. The Central Role of CD4(1) T Cells in the Antitumor Immune Response. J Exp Med (1998) 188:2357–68. 10.1084/jem.188.12.2357 PMC2212434 9858522

[52] Ishibashi S Ohashi Y Suzuki T Miyazaki S Moriya T Satomi S. Tumor-Associated Tissue Eosinophilia in Human Esophageal Squamous Cell Carcinoma. Anticancer Res (2006) 26:1419–24. 16619553

[53] Goldsmith MM Belchis DA Cresson DH Merritt WD, III Askin FB. The Importance of the Eosinophil in Head and Neck Cancer. Otolaryngol Head Neck Surg (1992) 106:27–33. 10.1177/019459989210600124 1734363

[54] Iwasaki K Torisu M Fujimura T. Malignant Tumor and Eosinophils I. Prognostic Significance in Gastric Cancer. Cancer (1986) 58:1321–7. 10.1002/1097-0142(19860915)58:6<1321::AID-CNCR2820580623>3.0.CO;2-O 3742457

[55] Liu M Kuo F Capistrano KJ Kang D Nixon BG Shi W. Tgf-β Suppresses Type 2 Immunity to Cancer. Nature (2020) 587(7832):115–20. 10.1038/s41586-020-2836-1 PMC8347705 33087928

[56] Lorvik KB Hammarstrom C Fauskanger M Haabeth OA Zangani M Haraldsen G. Adoptive Transfer of Tumor-Specific Th2 Cells Eradicates Tumors by Triggering an in Situ Inflammatory Immune Response. Cancer Res (2016) 76(23):6864–76. 10.1158/0008-5472.CAN-16-1219 27634753

[57] Modesti A Masuelli L Modica A D’Orazi G Scarpa S Bosco MC. Ultrastructural Evidence of the Mechanisms Responsible for Interleukin-4-Activated Rejection of a Spontaneous Murine Adenocarcinoma. Int J Cancer (1993) 53:988–93. 10.1002/ijc.2910530622 8473057

[58] Musiani P Allione A Modica A Lollini PL Giovarelli M Cavallo F. Role of Neutrophils and Lymphocytes in Inhibition of a Mouse Mammary Adenocarcinoma Engineered to Release IL-2, Il-4, IL-7, Il-10. IFN-Alpha, IFN-Gamma, and TNF-Alpha. Lab Invest (1996) 74:146–57. 10.1002/eji.201948336 8569177

[59] Nielsen HJ Hansen U Christensen IJ Reimert CM Brunner N Moesgaard F. Independent Prognostic Value of Eosinophil and Mast Cell Infiltration in Colorectal Cancer Tissue. J Pathol (1999) 189:487–95. 10.1002/(SICI)1096-9896(199912)189:4<487::AID-PATH484>3.0.CO;2-I 10629548

[60] Fernandez-Acenero MJ Galindo-Gallego M Sanz J Aljama A. Prognostic Influence of Tumor-Associated Eosinophilic Infiltrate in Colorectal Carcinoma. Cancer (2000) 88:1544–8. 10.1002/(SICI)1097-0142(20000401)88:7<1544::AID-CNCR7>3.0.CO;2-S 10738211

[61] Nishimura T Iwakabe K Sekimoto M Ohmi Y Yahata T Nakui M. Distinct Role of Antigen-Specific T Helper Type 1 (Th1) and Th2 Cells in Tumor Eradication In Vivo. J Exp Med (1999) 190:617–27. 10.1084/jem.190.5.617 PMC2195611 10477547

[62] Pericle F Giovarelli M Colombo MP Ferrari G Musiani P Modesti A. an Efficient Th2-Type Memory Follows CD81 Lymphocyte-Driven and Eosinophil-Mediated Rejection of a Spontaneous Mouse Mammary Adenocarcinoma Engineered to Release IL-4. J Immunol (1994) 153:5659–73. 7989764

[63] Reichman H Karo-Atar D Munitz A. Emerging Roles for Eosinophils in the Tumor Microenvironment. Trends Cancer (2016) 2(11):664–75. 10.1016/j.trecan.2016.10.002 28741505

[64] Reichman H Itan M Rozenberg P Yarmolovski T Brazowski E Varol C. Activated Eosinophils Exert Antitumorigenic Activities in Colorectal Cancer. Cancer Immunol Res (2019) 7(3):388–400. 10.1016/j.trecan.2016.10.002 30665890

[65] Tepper RI Coffman RL Leder P. An Eosinophil-Dependent Mechanism for the Antitumor Effect of Interleukin-4. Science (1992) 257:548–51. 10.1126/science.1636093 1636093

[66] Simson L Ellyard JI Dent LA Matthaei KI Rothenberg ME Foster PS. Regulation of Carcinogenesis by Interleukin-5 and CCL11: A Potential Role for Eosinophils in Tumour Immune Surveillance. J Immunol (2007) 178:4222–9. 10.4049/jimmunol.178.7.4222 17371978

[67] Wolf MT Ganguly S Wang TL Anderson CW Sadtler K Narain R. A Biologic Scaffold–Associated Type 2 Immune Microenvironment Inhibits Tumor Formation and Synergizes With Checkpoint Immunotherapy. Sci Transl Med (2019) 11:eaat7973. 10.1126/scitranslmed.aat7973 30700576 PMC7254933

[68] Aspord C Pedroza-Gonzalez A Gallegos M Tindle S Burton EC Su D. Breast Cancer Instructs Dendritic Cells to Prime Interleukin 13-Secreting CD4+ T Cells That Facilitate Tumor Development. J Exp Med (2007) 204(5):1037–47. 10.1084/jem.20061120 PMC2118566 17438063

[69] Chen J Gong C Mao H Li Z Fang Z Chen Q. H. Lie2f1/SP3/STAT6 Axis Is Required for IL-4-Induced Epithelial-Mesenchymal Transition of Colorectal Cancer Cells. Int J Oncol (2018) 53(2):567–78. 10.3892/ijo.2018.4429 PMC6017240 29901191

[70] Conticello C Pedini F Zeuner A Patti M Zerilli M Stassi G. IL-4 Protects Tumor Cells From Anti-CD95 and Chemotherapeutic Agents Via Up-Regulation of Antiapoptotic Proteins. J Immunol (2004) 172:5467–77. 10.4049/jimmunol.172.9.5467 15100288

[71] Dai M Hellstrom I Yip YY Sjögren HO Hellstrom KE. Tumor Regression and Cure Depends on Sustained Th1 Responses. J Immunother (2018) 41(8):369–78. 10.1097/CJI.0000000000000231 PMC6133214 29912725

[72] Hellstrom KE Dai M Hellstrom I. Curing Tumor-Bearing Mice by Shifting a Th2 to a Th1 Anti-Tumor Response. Hum Antibodies (2017) 25(3-4):147–53. 10.3233/HAB-160309 28085017

[73] De Monte L Reni M Tassi E Clavenna D Papa I Recalde H. Intratumor T Helper Type 2 Cell Infiltrate Correlates With Cancer-Associated Fibroblast Thymic Stromal Lymphopoietin Production and Reduced Survival in Pancreatic Cancer. J Exp Med (2011) 208:469–78. 10.1084/jem.20101876 PMC3058573 21339327

[74] DeNardo DG Barreto JB Andreu P Vasquez L Tawfik D Kolhatkar N. Cd4(+) T Cells Regulate Pulmonary Metastasis of Mammary Carcinomas by Enhancing Protumor Properties of Macrophages. Cancer Cell (2009) 16(2):91–102. 10.1016/j.ccr.2009.06.018 19647220 PMC2778576

[75] Espinoza JA Jabeen S Batra R Papaleo E Haakensen V Timmermans Wielenga V. Cytokine Profiling of Tumour Interstitial Fluid of the Breast and Its Relationship With Lymphocyte Infiltration and Clinicopathological Characteristics. Oncoimmunology (2016) 5:00–0. 10.1080/2162402X.2016.1248015 PMC5215081 28123884

[76] Feng Q Wei H Morihara J. Th2 Type Inflammation Promotes the Gradual Progression of HPV-Infected Cervical Cells to Cervical Carcinoma. Gynecol Oncol (2012) 127(2):412–9. 10.1016/j.ygyno.2012.07.098 PMC3472044 22828962

[77] Gurusamy D Shoe J Hocker J Hurwitz A. A Role for IL-13 in the Progression of Prostate Tumors (TUM10P.1046). J Immunol (2015) 194(1 Supplement):211–27.

[78] Prokopchuk O Liu Y Henne-Bruns D Kornmann M. Interleukin-4 Enhances Proliferation of Human Pancreatic Cancer Cells: Evidence for Autocrine and Paracrine Actions. Br J Cancer (2005) 92:921–8. 10.1038/sj.bjc.6602416 PMC2361902 15714203

[79] Suzuki A Leland P Joshi BH Puri RK. Targeting of IL-4 and IL-13 Receptors for Cancer Therapy. Cytokine (2015) 75:79–88. 10.1016/j.cyto.2015.05.026 26088753

[80] Terabe M Park JM Berzofsky JA. Role of IL-13 in Regulation of Anti-Tumor Immunity and Tumor Growth. Cancer Immunol Immunother (2004) 53:79–85. 10.1007/s00262-003-0445-0 14610620 PMC11034335

[81] Traub B Sun L Ma Y Xu P Lemke J Paschke S. Endogenously Expressed IL-4Ralpha Promotes the Malignant Phenotype of Human Pancreatic Cancer In Vitro and In Vivo. Int J Mol Sci (2017) 18(4):716. 10.3390/ijms18040716 PMC5412302 28350325

[82] Tokumaru Y Le L Oshi M Katsuta E Matsuhashi N Futamura M. Association of Th2 High Tumors With Aggressive Features of Breast Cancer. J Clin Oncol (2020) 38(15_suppl):e12584. 10.1200/JCO.2020.38.15_suppl.e12584

[83] Zaynagetdinov R Sherrill TP Gleaves LA McLoed AG Saxon JA Habermann AC. Interleukin-5 Facilitates Lung Metastasis by Modulating the Immune Microenvironment. Cancer Res (2015) 75:1624–34. 10.1158/0008-5472.CAN-14-2379 PMC4401663 25691457

[84] Zhang Q Qin J Zhong L Gong L Zhang B Zhang Y. CCL5-Mediated Th2 Immune Polarization Promotes Metastasis in Luminal Breast Cancer. Cancer Res (2015) 75(20):4312–21. 10.1158/0008-5472.CAN-14-3590 26249173

[85] Spranger S. Mechanisms of Tumor Escape in the Context of the T-Cell-Inflamed and the Non-T-Cell-Inflamed Tumor Microenvironment. Int Immunol (2016) 28(8):383–91. 10.1093/intimm/dxw014 PMC4986232 26989092

[86] Zhang C Yue C Herrmann A Song J Egelston C Wang T. Stat3 Activation-Induced Fatty Acid Oxidation in CD8+ T Effector Cells Is Critical for Obesity-Promoted Breast Tumor Growth. Cell Metab (2020) 31(1):148–61.e5. 10.1016/j.cmet.2019.10.013 31761565 PMC6949402

[87] Fischer K Hoffmann P Voelkl S Meidenbauer N Ammer J Edinger M. Inhibitory Effect of Tumor Cell-Derived Lactic Acid on Human T Cells. Blood (2007) 109(9):3812–9. 10.1182/blood-2006-07-035972 17255361

[88] DuPage M Cheung AF Mazumdar C Winslow MM Bronson R Schmidt LM. Endogenous T Cell Responses to Antigens Expressed in Lung Adenocarcinomas Delay Malignant Tumor Progression. Cancer Cell (2011) 19(1):72–85. 10.1016/j.ccr.2010.11.011 21251614 PMC3069809

[89] Neill DR Wong SH Bellosi A Flynn RJ Daly M Langford TK. Nuocytes Represent a New Innate Effector Leukocyte That Mediates Type-2 Immunity. Nature (2010) 464:1367–70. 10.1038/nature08900 PMC2862165 20200518

[90] Mjösberg J Bernink J Golebski K Karrich JJ Peters CP Blom B. The Transcription Factor GATA3 Is Essential for the Function of Human Type 2 Innate Lymphoid Cells. Immunity (2012) 37(4):649–59. 10.1016/j.immuni.2012.08.015 23063330

[91] Moro K Yamada T Tanabe M Takeuchi T Ikawa T Kawamoto H. Innate Production of T(H)2 Cytokines by Adipose Tissue-Associated C-Kit(+)Sca-1(+) Lymphoid Cells. Nature (2010) 463:540–4. 10.1038/nature08636 20023630

[92] Saranchova I Han J Zaman R Arora H Huang H Fenninger F. Type 2 Innate Lymphocytes Actuate Immunity Against Tumours and Limit Cancer Metastasis. Sci Rep (2018) 8(1):2924. 10.1038/s41598-018-20608-6 29440650 PMC5811448

[93] Moral JA Leung J Rojas LA Ruan J Zhao J Sethna Z. ILC2s Amplify PD-1 Blockade by Activating Tissue-Specific Cancer Immunity. Nature (2020) 579(7797):130–5. 10.1038/s41586-020-2015-4 PMC7060130 32076273

[94] Kim J Kim W Moon UJ Kim HJ Choi HJ Sin JI. Intratumorally Establishing Type 2 Innate Lymphoid Cells Blocks Tumor Growth. J Immunol (2016) 196:2410–23. 10.4049/jimmunol.1501730 26829987

[95] Ercolano G Falquet M Vanoni G Trabanelli S Jandus C. Ilc2s: New Actors in Tumor Immunity. Front Immunol (2019) 10:2801. 10.3389/fimmu.2019.02801 31849977 PMC6902088

[96] Liu M Kuo F KJ C Kang D BG N Shi W. TGF-Beta Suppresses Type 2 Immunity to Cancer. Nature (2020) 587(7832):115–20. 10.1038/s41586-020-2836-1 PMC8347705 33087928

[97] Ribatti D. Mast Cells and Macrophages Exert Beneficial and Detrimental Effects on Tumor Progression and Angiogenesis. Immunol Lett (2013) 152(2):83–8. 10.1016/j.imlet.2013.05.003 23685256

[98] Purwar R Schlapbach C Xiao S Kang HS Elyaman W Jiang X. Robust Tumor Immunity to Melanoma Mediated by Interleukin-9-Producing T Cells. Nat Med (2012) 18(8):1248–53. 10.1038/nm.2856 PMC3518666 22772464

[99] Liu J-Q Li X-Y Yu H-Q Yang G Liu Z-Q Geng X-R. Tumor-Specific Th2 Responses Inhibit Growth of CT26 Colon-Cancer Cells in Mice Via Converting Intratumor Regulatory T Cells to Th9 Cells. Sci Rep (2015) 5:10665. 10.1038/srep10665 26035423 PMC4451845

[100] Baumeister SH Freeman GJ Dranoff G Sharpe AH. Epub 2016 Feb 25. Coinhibitory Pathways in Immunotherapy for Cancer. Annu Rev Immunol (2016) 34:539–73. 10.1146/annurev-immunol-032414-112049 26927206

[101] June CH O’Connor RS Kawalekar OU Ghassemi S Milone MC. Car T Cell Immunotherapy for Human Cancer. Science (2018) 359(6382):1361–5. 10.1126/science.aar6711 29567707

[102] Cervantes-Villagrana RD Albores-García D Cervantes-Villagrana AR García-Acevez SJ. Tumor-Induced Neurogenesis and Immune Evasion as Targets of Innovative Anti-Cancer Therapies. Signal Transduct Target Ther (2020) 5(1):99. 10.1038/s41392-020-0205-z 32555170 PMC7303203

[103] Le Bourgeois T Strauss L Aksoylar H-I Daneshmandi S Seth P Patsoukis N. Targeting T Cell Metabolism for Improvement of Cancer Immunotherapy. Front Oncol (2018) 8:237. 10.3389/fonc.2018.00237 30123774 PMC6085483

[104] Munn DH Mellor AL. Indoleamine 2,3 Dioxygenase and Metabolic Control of Immune Responses. Trends Immunol (2013) 34(3):137–43. 10.1016/j.it.2012.10.001 PMC3594632 23103127

[105] DeBerardinis RJ Chandel NS. Fundamentals of Cancer Metabolism. Sci Adv (2016) 2(5):e1600200. 10.1126/sciadv.1600200 27386546 PMC4928883

[106] Potter M Newport E Morten KJ The Warburg Effect: 80 Years on. Biochem Soc Trans (2016) 44(5):1499–505. 10.1042/BST20160094 PMC5095922 27911732

[107] Sullivan MR Danai LV Lewis CA Chan SH Gui DY Kunchok T. Quantification of Microenvironmental Metabolites in Murine Cancers Reveals Determinants of Tumor Nutrient Availability. Elife (2019) 8:e44235. 10.7554/eLife.44235 30990168 PMC6510537

[108] Kaymak I Williams KS Cantor JR Jones RG. Online Ahead of Print. Immunometabolic Interplay in the Tumor Microenvironment. Cancer Cell (2021) 39(1):28–37. 10.1016/j.ccell.2020.09.004 33125860 PMC7837268

[109] Kim SY. Targeting Cancer Energy Metabolism: A Potential Systemic Cure for Cancer. Arch Pharm Res (2019) 42:140–9. 10.1007/s12272-019-01115-2 30656605

[110] Vander Heiden MG Plas DR Rathmell JC Fox CJ Harris MH Thompson CB. Growth Factors can Influence Cell Growth and Survival Through Effects on Glucose Metabolism. Mol Cell Biol (2001) 21(17):5899–912. 10.1128/mcb.21.17.5899-5912.2001 PMC87309 11486029

[111] Buchakjian MR Kornbluth S. The Engine Driving the Ship: Metabolic Steering of Cell Proliferation and Death. Nat Rev Mol Cell Biol (2010) 11(10):715–27. 10.1038/nrm2972 20861880

[112] Ho P-C Bihuniak JD Macintyre AN Staron M Liu X Amezquita R. Phosphoenolpyruvate Is a Metabolic Checkpoint of Anti-Tumor T Cell Responses. Cell (2015) 162:1217–28. 10.1016/j.cell.2015.08.012 PMC4567953 26321681

[113] Brand A Singer K Koehl GE Kolitzus M Schoenhammer G Thiel A. Ldha-Associated Lactic Acid Production Blunts Tumor Immunosurveillance by T and NK Cells. Cell Metab (2016) 24(5):657–71. 10.1016/j.cmet.2016.08.011 27641098

[114] Vaeth M Maus M Klein-Hessling S Freinkman E Yang J Eckstein M. Store-Operated Ca 2+ Entry Controls Clonal Expansion of T Cells Through Metabolic Reprogramming. Immunity (2017) 47(4):664–79.e6. 10.1016/j.immuni.2017.09.003 29030115 PMC5683398

[115] Angelin A Gil-de-Gómez L Dahiya S Jiao J Guo L Levine MH. Fp3ox Reprograms T Cell Metabolism to Function in Low-Glucose, High-Lactate Environments. Cell Metab (2017) 25:1282–93.e7. 10.1016/j.cmet.2016.12.018 28416194 PMC5462872

[116] Wang H Franco F Tsui YC Xie X Trefny MP Zappasodi R. CD36-Mediated Metabolic Adaptation Supports Regulatory T Cell Survival and Function in Tumors. Nat Immunol (2020) 21:298–308. 10.1038/s41590-019-0589-5 32066953 PMC7043937

[117] Field CS Baixauli F Kyle RL Puleston DJ Cameron AM Sanin DE. Mitochondrial Integrity Regulated by Lipid Metabolism Is a Cell-Intrinsic Checkpoint for Treg Suppressive Function. Cell Metab (2020) 31(2):422–437.e5. 10.1016/j.cmet.2019.11.021 31883840 PMC7001036

[118] Ren W Liu G Yin J Tan B Wu G Bazer FW. Amino-Acid Transporters in T-Cell Activation and Differentiation. Cell Death Dis (2017) 8(3):e2655. 10.1038/cddis.2016.222 28252650 PMC5386510

[119] Lukey MJ Katt WP Cerione RA. Targeting Amino Acid Metabolism for Cancer Therapy. Drug Discovery Today (2017) 22(5):796–804. 10.1016/j.drudis.2016.12.003 27988359 PMC5429979

[120] Pan M Reid MA Lowman XH Kulkarni RP Tran TQ Liu X. Regional Glutamine Deficiency in Tumours Promotes Dedifferentiation Through Inhibition of Histone Demethylation. Nat Cell Biol (2016) 18:1090–101. 10.1038/ncb3410 PMC5536113 27617932

[121] Howden AJM Hukelmann JL Brenes A Spinelli L Sinclair LV Lamond AI. Quantitative Analysis of T Cell Proteomes and Environmental Sensors During T Cell Differentiation. Nat Immunol (2019) 20(11):1542–54. 10.1038/s41590-019-0495-x PMC6859072 31591570

[122] Ackerman D Simon MC. Hypoxia, Lipids, and Cancer: Surviving the Harsh Tumor Microenvironment. Trends Cell Biol (2014) 24(8):472–8. 10.1016/j.tcb.2014.06.001 PMC4112153 24985940

[123] Corn KC Windham MA Rafat M. Lipids in the Tumor Microenvironment: From Cancer Progression to Treatment. Prog Lipid Res (2020) 80:101055. 10.1016/j.plipres.2020.101055 32791170 PMC7674189

[124] Hollander DM Ebert EC Roberts AI Devereux DF. Effects of Tumor Type and Burden on Carcass Lipid Depletion in Mice. D M Hollander. Surgery (1986) 100(2):292–7. 3738756

[125] Zhang M Martino JSD Bowman RL Campbell NR Baksh SC Simon-Vermot T. Adipocyte-Derived Lipids Mediate Melanoma Progression Via Fatp Proteins. Cancer Discovery (2018) 8(8):1006–25. 10.1158/1538-7445.AM2018-5120 PMC6192670 29903879

[126] Wang YY Attané C Milhas D Dirat B Dauvillier S Guerard A. Mammary Adipocytes Stimulate Breast Cancer Invasion Through Metabolic Remodeling of Tumor Cells. JCI Insight (2017) 2(4):e87489. 10.1172/jci.insight.87489 PMC5313068 28239646

[127] Balaban S Shearer RF Lee LS van Geldermalsen M Schreuder M Shtein HC. Adipocyte Lipolysis Links Obesity to Breast Cancer Growth: Adipocyte-Derived Fatty Acids Drive Breast Cancer Cell Proliferation and Migration. Cancer Metab (2017) 5:1. 10.1186/s40170-016-0163-7 28101337 PMC5237166

[128] Nieman KM Kenny HA Penicka CV Ladanyi A Buell-Gutbrod R Zillhardt MR. Adipocytes Promote Ovarian Cancer Metastasis and Provide Energy for Rapid Tumor Growth. Nat Med (2011) 17(11):1498–503. 10.1038/nm.2492 PMC4157349 22037646

[129] Gazi E Gardner P Lockyer NP Hart CA Brown MD Clarke NW. Direct Evidence of Lipid Translocation Between Adipocytes and Prostate Cancer Cells With Imaging FTIR Microspectroscopy. J Lipid Res (2007) 48(8):1846–56. 10.1194/jlr.M700131-JLR200 17496269

[130] Manzo T Prentice BM Anderson KG Raman A Schalck A Codreanu GS. Accumulation of Long-Chain Fatty Acids in the Tumor Microenvironment Drives Dysfunction in Intrapancreatic CD8+ T Cells. J Exp Med (2020) 217:e201919202020. 10.1084/jem.20191920 PMC7398173 32491160

[131] Eil R Vodnala SK Clever D Klebanoff CA Sukumar M Pan JH. Ionic Immune Suppression Within the Tumour Microenvironment Limits T Cell Effector Function. Nature (2016) 537(7621):539–43. 10.1038/nature19364 PMC5204372 27626381

[132] Weidinger C Shaw PJ Feske S. STIM1 and STIM2-Mediated Ca2+ Influx Regulates Antitumour Immunity by CD8+ T Cells. EMBO Mol Med (2013) 5(9):1311–21. 10.1002/emmm.201302989 PMC3799488 23922331

[133] Kleinewietfeld M Manzel A Titze J Kvakan H Yosef N Linker RA. Sodium Chloride Drives Autoimmune Disease by the Induction of Pathogenic Th17 Cells. Nature (2013) 496(7446):518–22. 10.1038/nature11868 PMC3746493 23467095

[134] Matthias J Heink S Picard F Zeiträg J Kolz A Chao Y-Y. Salt Generates Antiinflammatory Th17 Cells But Amplifies Pathogenicity in Proinflammatory Cytokine Microenvironments. J Clin Invest (2020) 130(9):4587–600. 10.1172/JCI137786 PMC7456214 32484796

[135] Kaufmann U Kahlfuss S Yang J Ivanova E Koralov SB Feske S. Calcium Signaling Controls Pathogenic Th17 Cell-Mediated Inflammation by Regulating Mitochondrial Function. Cell Metab (2019) 29(5):1104–18.e6. 10.1016/j.cmet.2019.01.019 30773462 PMC6506368

[136] Kahlfuss S Kaufmann U Concepcion AR Noyer L Raphael D Vaeth M. STIM1-Mediated Calcium Influx Controls Antifungal Immunity and the Metabolic Function of Non-Pathogenic Th17 Cells. EMBO Mol Med (2020) 12(8):e11592. 10.15252/emmm.201911592 32609955 PMC7411566

[137] Bertout JA Patel SA Simon MC. The Impact of O2 Availability on Human Cancer. Nat Rev Cancer (2008) 8:967–75. 10.1038/nrc2540 PMC3140692 18987634

[138] Gropper Y Feferman T Shalit T Salame T-M Porat Z Shakhar G. Culturing Ctls Under Hypoxic Conditions Enhances Their Cytolysis and Improves Their Anti-Tumor Function. Cell Rep (2017) 20:2547–55. 10.1016/j.celrep.2017.08.071 28903036

[139] Papandreou I Cairns RA Fontana L Lim AL Denko NC. HIF-1 Mediates Adaptation to Hypoxia by Actively Downregulating Mitochondrial Oxygen Consumption. Cell Metab (2006) 3:187–97. 10.1016/j.cmet.2006.01.012 16517406

[140] Kim JW Tchernyshyov I Semenza GL Dang CV. Hif-1-Mediated Expression of Pyruvate Dehydrogenase Kinase: A Metabolic Switch Required for Cellular Adaptation to Hypoxia. Cell Metab (2006) 3:177–85. 10.1016/j.cmet.2006.02.002 16517405

[141] Clever D Roychoudhuri R Constantinides MG Askenase MH Sukumar M Klebanoff CA. Oxygen Sensing by T Cells Establishes an Immunologically Tolerant Metastatic Niche. Cell (2016) 166(5):1117–1131.e14. 10.1016/j.cell.2016.07.032 27565342 PMC5548538

[142] Noman MZ Desantis G Janji B Hasmim M Karray S Dessen P. Pd-L1 Is a Novel Direct Target of HIF-1a, and Its Blockade Under Hypoxia Enhanced MDSC-Mediated T Cell Activation. J Exp Med (2014) 211:781–90. 10.1084/jem.20131916 PMC4010891 24778419

[143] Ben-Shoshan J Maysel-Auslender S Mor A Keren G George J. Hypoxia Controls CD4+CD25+ Regulatory T-Cell Homeostasis Via Hypoxia-Inducible Factor-1alpha. Eur J Immunol (2008) 38:2412–8. 10.1002/eji.200838318 18792019

[144] Zhang Y Kurupati R Liu L Zhou XY Zhang G Hudaihed A. Author Manuscript; Available in PMC 2018 Sep 11. Enhancing Cd8+ T Cell Fatty Acid Catabolism Within a Metabolically Challenging Tumor Microenvironment Increases the Efficacy of Melanoma Immunotherapy. Cancer Cell (2017) 32(3):377–91.e9. 10.1016/j.ccell.2017.08.004 PMC5751418 28898698

[145] Hornyák L Dobos N Koncz G Karányi Z Páll D Szabó Z. The Role of Indoleamine-2,3-Dioxygenase in Cancer Development, Diagnostics, and Therapy. Front Immunol (2018) 9:151. 10.3389/fimmu.2018.00151 29445380 PMC5797779

[146] Labani-Motlagh A Ashja-Mahdavi M Loskog A. The Tumor Microenvironment: A Milieu Hindering and Obstructing Antitumor Immune Responses. Front Immunol (2020) 11:940. 10.3389/fimmu.2020.00940 32499786 PMC7243284

[147] Tiwary S Berzofsky JA Terabe M. Altered Lipid Tumor Environment and Its Potential Effects on NKT Cell Function in Tumor Immunity. Front Immunol (2019) 10:2187. 10.3389/fimmu.2019.02187 31620124 PMC6759687

[148] Rongchen S Yi-Quan T Hongming M. Metabolism in Tumor Microenvironment: Implications for Cancer Immunotherapy. MedComm (2020) 1:47–68. 10.1002/mco2.6 PMC8489668 34766109

[149] Chang C-H Qiu J O’Sullivan D Buck MD Noguchi T Curtis JD. Metabolic Competition in the Tumor Microenvironment Is a Driver of Cancer Progression. Cell (2015) 162:1229–41. 10.1016/j.cell.2015.08.016 PMC4864363 26321679

[150] Pelgrom LR Everts B. Metabolic Control of Type 2 Immunity. Eur J Immunol (2017) 47(8):1266–75. 10.1002/eji.201646728 28661041

[151] Wilhelm C Harrison OJ Schmitt V Pelletier M Spencer SP Urban JF Jr.. Critical Role of Fatty Acid Metabolism in ILC2-Mediated Barrier Protection During Malnutrition and Helminth Infection. J Exp Med (2016) 213(8):1409–18. 10.1084/jem.20151448 PMC4986525 27432938

[152] Monami M Dicembrini I Mannucci E. Thiazolidinediones and Cancer: Results of a Meta-Analysis of Randomized Clinical Trials. Acta Diabetol (2014) 51(1):91–101. 10.1007/s00592-013-0504-8 23851465

[153] Liu Y Jin PP Sun XC Hu TT. Thiazolidinediones and Risk of Colorectal Cancer in Patients With Diabetes Mellitus: A Meta-Analysis. Saudi J Gastroenterol (2018) 24(2):75–81. 10.4103/sjg.SJG_295_17 29637913 PMC5900477

[154] Wuertz BR Darrah L Wudel J Ondrey FG. Thiazolidinediones Abrogate Cervical Cancer Growth. Exp Cell Res (2017) 353(2):63–71. 10.1016/j.yexcr.2017.02.020 28219679

[155] Blanquicett C Roman J Hart CM. Thiazolidinediones as Anti-Cancer Agents. Cancer Ther (2008) 6(A):25–34. 19079765 PMC2600565

[156] Cipolletta D Feuerer M Li A Kamei N Lee J Shoelson SE. Ppar-γ Is a Major Driver of the Accumulation and Phenotype of Adipose Tissue Treg Cells. Nature (2012) 486(7404):549–53. 10.1038/nature11132 PMC3387339 22722857

[157] Farhadi P Yarani R Dokaneheifard S Mansouri K. The Emerging Role of Targeting Cancer Metabolism for Cancer Therapy. Tumour Biol (2020) 42(10):1010428320965284. 10.1177/1010428320965284 33028168

[158] Luengo A Gui DY Vander Heiden MG. Targeting Metabolism for Cancer Therapy. Cell Chem Biol (2017) 24(9):1161–80. 10.1016/j.chembiol.2017.08.028 PMC5744685 28938091

[159] Kouidhi S Ben Ayed F Benammar Elgaaied A. Targeting Tumor Metabolism: A New Challenge to Improve Immunotherapy. Front Immunol (2018) 9:353. 10.3389/fimmu.2018.00353 29527212 PMC5829092

[160] Yang M Ma C Liu S Sun J Shao Q Gao W. Hypoxia Skews Dendritic Cells to a T Helper Type 2-Stimulating Phenotype and Promotes Tumour Cell Migration by Dendritic Cell-Derived Osteopontin. Immunology (2009) 128:e237–49. 10.1111/j.1365-2567.2008.02954.x PMC2753916 19740309

[161] Yang M Ma C Liu S Shao Q Gao W Song B. HIF-Dependent Induction of Adenosine Receptor A2b Skews Human Dendritic Cells to a Th2-Stimulating Phenotype Under Hypoxia. Immunol Cell Biol (2010) 88:165–71. 10.1038/icb.2009.77 19841638

[162] Goda N Kanai M. Hypoxia-Inducible Factors and Their Roles in Energy Metabolism. Int J Hematol (2012) 95(5):457–63. 10.1007/s12185-012-1069-y 22535382

[163] Miska J Lee-Chang C Rashidi A Muroski ME Chang AL Lopez-Rosas A. Hif-1α Is a Metabolic Switch Between Glycolytic-Driven Migration and Oxidative Phosphorylation-Driven Immunosuppression of Tregs in Glioblastoma. Cell Rep (2019) 27(1):226–37.e4. 10.1016/j.celrep.2019.03.029 30943404 PMC6461402

[164] Cho SH Raybuck AL Blagih J Kemboi E Haase VH Jones RG. Hypoxia-Inducible Factors in CD4+ T Cells Promote Metabolism, Switch Cytokine Secretion, and T Cell Help in Humoral Immunity. Proc Natl Acad Sci USA (2019) 116:8975–898. 10.1073/pnas.1811702116 PMC6500120 30988188

[165] Shi LZ Wang R Huang G Vogel P Neale G Green DR. HIF1alpha-Dependent Glycolytic Pathway Orchestrates a Metabolic Checkpoint for the Differentiation of TH17 and Treg Cells. J Exp Med (2011) 208(7):1367–76. 10.1084/jem.20110278 PMC3135370 21708926

[166] Yong CSM Dardalhon V Devaud C Taylor N Darcy PK Kershaw MH. CAR T-Cell Therapy of Solid Tumors. Immunol Cell Biol (2017) 95:356–63. 10.1038/icb.2016.128 28003642

[167] Knochelmann HM Smith AS Dwyer CJ Wyatt MM Mehrotra S Paulos CM. Car T Cells in Solid Tumors: Blueprints for Building Effective Therapies. Front Immunol (2018) 9:1740. 10.3389/fimmu.2018.01740 30140266 PMC6094980

[168] Fu C Jiang A. Dendritic Cells and CD8 T Cell Immunity in Tumor Microenvironment. Front Immunol (2018) 9:3059. 10.3389/fimmu.2018.03059 30619378 PMC6306491

[169] Lee GK Park HJ Macleod M Chandler P Munn DH Mellor AL. Tryptophan Deprivation Sensitizes Activated T Cells to Apoptosis Prior to Cell Division. Immunology (2002) 107(4):452–60. 10.1046/j.1365-2567.2002.01526.x PMC1782830 12460190

[170] Liu M Wang X Wang L Ma X Gong Z Zhang S. Targeting the IDO1 Pathway in Cancer: From Bench to Bedside. Hematol Oncol (2018) 11(1):100. 10.1186/s13045-018-0644-y PMC6090955 30068361

[171] Mezrich JD Fechner JH Zhang X Johnson BP Burlingham WJ Bradfield CA. An Interaction Between Kynurenine and the Aryl Hydrocarbon Receptor can Generate Regulatory T Cells. J Immunol (2010) 185(6):3190–8. 10.4049/jimmunol.0903670 PMC2952546 20720200

[172] Opitz C Litzenburger U Sahm F Ott M Tritschler I Trump S. An Endogenous Tumour-Promoting Ligand of the Human Aryl Hydrocarbon Receptor. Nature (2011) 478:197–203. 10.1038/nature10491 21976023

[173] Quintana F Basso A Iglesias A Korn T Farez MF Bettelli E. Control of Treg and TH17 Cell Differentiation by the Aryl Hydrocarbon Receptor. Nature (2008) 453:65–71. 10.1038/nature06880 18362915

[174] Le Naour J Galluzzi L Zitvogel L Kroemer G Vacchelli E. Trial Watch: IDO Inhibitors in Cancer Therapy. OncoImmunology (2014) 3(10):e957994. 10.1080/2162402X.2020.1777625 25941578 PMC4292223

[175] Vaeth M Kahlfuss S Feske S. Crac Channels and Calcium Signaling in T Cell-Mediated Immunity. Trends Immunol (2020) 41(10):878–901. 10.1016/j.it.2020.06.012 32711944 PMC7985820

[176] Panyi G Vámosi G Bacsó Z Bagdány M Bodnár A Varga Z. Kv1.3 Potassium Channels Are Localized in the Immunological Synapse Formed Between Cytotoxic and Target Cells. Proc Natl Acad Sci USA (2004) 101(5):1285–90. 10.1073/pnas.0307421100 PMC337045 14745040

